# Understanding the Barriers and Benefits to Scholarly Writing Among Internal Medicine Residents: A Pilot Study of an Innovative Education Method

**DOI:** 10.7759/cureus.58817

**Published:** 2024-04-23

**Authors:** Sumugdha Rayamajhi, Amey Joshi, Ling Wang, Ayushma Duwadi

**Affiliations:** 1 Internal Medicine, Michigan State University College of Human Medicine, East Lansing, USA; 2 Internal Medicine, Sparrow Health System, Lansing, USA; 3 Medicine, Michigan State University, East Lansing, USA; 4 Internal Medicine, Michigan State University, East Lansing, USA

**Keywords:** publications, resident training, case report, resident education, scholarly work

## Abstract

Background

This study addresses the participation gap in scholarly activities among internal medicine residents. While the Accreditation Council for Graduate Medical Education emphasizes the importance of these activities, the variability in their definition and support across residency programs presents a challenge. This study investigates these discrepancies and aims to identify the specific barriers and benefits residents perceive in scholarly writing, especially in case report writing, and to propose effective educational interventions.

Methodology

A voluntary online survey, pre- and post-educational intervention, was conducted among residents at Sparrow Hospital, Michigan State University. The intervention comprised a two-hour session focusing on case report writing, presentation skills, scientific literature searches, and research project engagement. Responses were obtained on a five-point Likert scale, and the data were analyzed as respective frequencies and percentages.

Results

Of 45 residents, 23 completed the pre-survey. With a response rate of 51% from the internal medicine residents, the post-intervention data revealed considerable improvement in the residents’ understanding and appreciation of scholarly activities. There was a marked enhancement in their skills related to scientific literature search and in recognizing the benefits of scholarly engagement. Additionally, the intervention successfully increased their confidence in presenting scholarly work, networking, and identifying relevant venues for their research.

Conclusions

The study highlights the challenges residents face in scholarly activities, such as lack of training and mentorship. It suggests that focused workshops and mentorship can significantly enhance residents’ research skills and confidence.

## Introduction

Residents in training programs are expected to participate in scholarly activities per Accreditation Council for Graduate Medical Education (ACGME) requirements [1]. The activities include presentations and publications of case reports, original research, and quality improvement projects. Residents in all years of training are expected to learn basic research principles and apply the relevant findings in clinical practice. However, due to the need for a standard definition for scholarly activities in ACGME, every residency program adopts unique ways to engage its residents. Despite these initiatives, lack of research curriculum training, faculty mentorship, financial assistance, and time are commonly cited barriers to scholarly work [2,3].

Case report writing can promote scientific discovery and serve as an educational tool to enhance critical thinking and presentation skills [2,3]. We conducted a voluntary online survey among 45 internal medicine residents to better understand their perception of and barriers to scholarly writing, primarily focussed on case report writing. Furthermore, after the pre-survey, we conducted a two-hour-long session addressing the benefits and obstacles to scholarly writing. The session not only addressed barriers to case report writing but also facilitated techniques in making effective poster and oral presentations, performing effective scientific literature searches, and utilizing resources and opportunities to engage in research projects.

## Materials and methods

A voluntary online pre- and post-Qualtrix survey was conducted among internal medicine residents at Sparrow Hospital, affiliated with Michigan State University. Study approval was obtained from the Michigan State University Institutional Review Board (approval number: STUDY00010062), ensuring adherence to ethical guidelines. Participants were informed about the study’s nature through an email that included a link to the survey, and anonymity was maintained throughout the process.

Data collection and analysis were conducted using the Qualtrix online survey platform. The survey link was emailed to 45 first-year to third-year internal medicine residents. The survey questionnaire assessed the barriers to and benefits of case report writing before and after the intervention. Responses were collected using a five-point Likert scale (Table 1). Phase 1 involved the pre-survey questionnaire. During this phase, insights into barriers and benefits regarding case report writing were gathered. Phase 2 comprised the intervention, which involved a live interactive PowerPoint presentation by internal medicine faculties. Over two hours, the session addressed barriers to and benefits associated with scholarly writing. The session included a guided tutorial on creating effective posters and oral presentations and conducting scientific literature searches [4]. The session also covered the issues of citation chaining and utilizing resources such as PubMed, Medline, MeSH, and Cochrane while exploring opportunities for research projects. A teaching module from the Medical College of Wisconsin was used for additional resources. Trainees were encouraged and invited to use the library portal, article request form, or interlibrary loan services that could help obtain articles that are not freely available. Trainees were encouraged to approach faculty, peers, and seniors for mentorship. Phase 3 encompassed the post-survey to evaluate the usefulness and comprehension of the interactive session. By providing support and resources through interactive sessions, we aimed to assist residents in fulfilling their residency program requirements, enhancing their academic careers, and nurturing intellectual curiosity.

**Table 1:** Pre- and post-survey questionnaire. Adapted from Tumilty et al. [3].

Q1. Factors that facilitate writing and presenting case reports
1. Finding an interesting case
2. Finding a good mentor
3. Having financial assistance
4. Lectures and workshops
Q2. Benefits of writing and presenting case reports
1. Improves presentation skills
2. Improves scientific writing skills
3. Enhances CV and secures fellowship position
4. Improves critical thinking
5. Networking and collaboration
Q3. Barriers to writing and presenting case reports
1. Lack of training in reviewing scientific literature
2. Lack of adequate time during residency
3. Lack of formal training in identifying and writing case reports
4. Lack of mentor(s)
5. Lack of opportunities/venues to present
6. Lack of financial assistance

## Results

**Figure 1:**
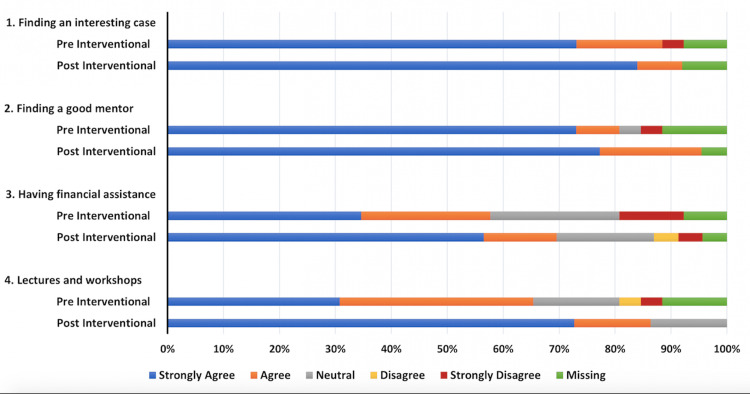
Factors that facilitate writing and presenting case reports. The data are represented as percentages in bar graphs.

**Figure 2:**
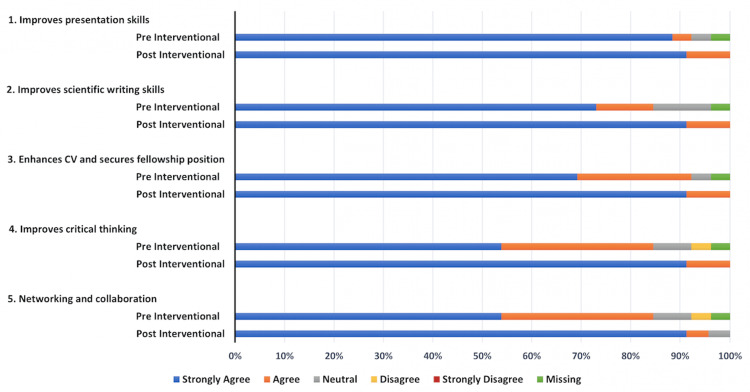
Benefits of writing and presenting case reports. The data are represented as percentages in bar graphs.

**Figure 3:**
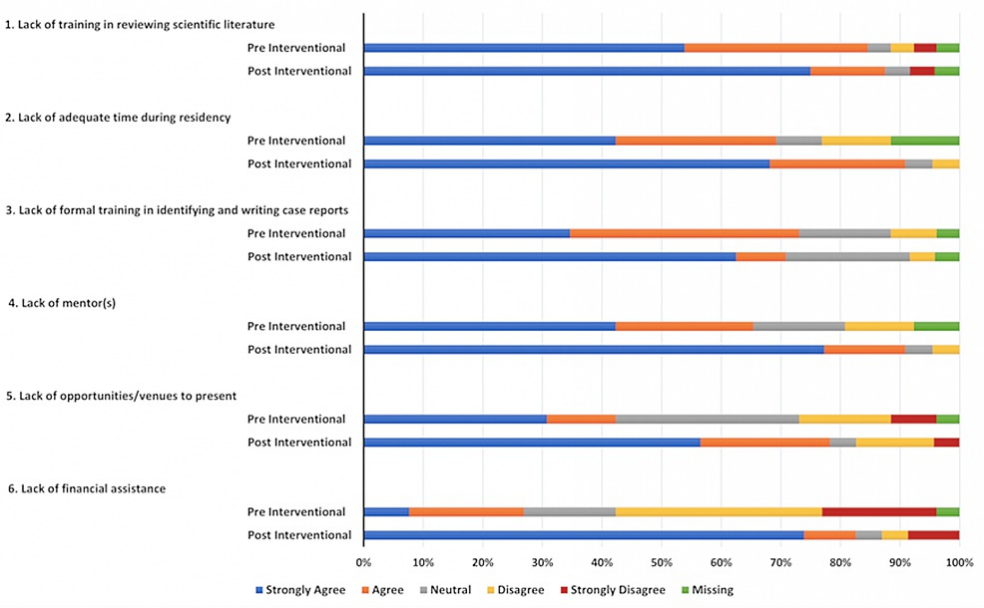
Barriers to writing and presenting case reports. The data are represented as percentages in bar graphs.

Of the 45 internal medicine residents, 23 completed the pre-survey with a response rate of 51%. The ordered probit regression model was used for ordinal dependent variables to assess and compare outcomes. The cutoff for the p-value was <0.05. Following a brief, targeted intervention consisting of lectures and workshop (phase 2), trainees exhibited a significant improvement in their comprehension of the factors that aid in writing and presenting scholarly activity, as evidenced by a higher score compared to pre-intervention (odds ratio (OR) = 4.25, 95% confidence interval (CI) = 1.27-14.22, p = 0.02) (Figure 1). Moreover, trainees demonstrated a heightened perception of the benefits of scholarly activity, with a notable increase in the belief that such engagement enhances their critical thinking skills (OR = 8.578, 95% CI = 1.639-44.895, p = 0.02) (Figure 2). Trainees perceived networking and collaboration to be a significant advantage of presenting, a perception notably strengthened following the intervention (OR = 7.78, 95% CI = 1.51-39.94, p = 0.01) (Figure 2). The initial barrier of inadequate mentorship for case writing and presentation was effectively addressed in the intervention session. This session illustrated how mentorship can extend beyond faculty to include peers and seniors, leading to a notable improvement in post-intervention outcomes related to scholarly activities (OR = 3.985, 95% CI = 1.143-13.8, p = 0.03) (Figure 2). The lack of training in reviewing scientific literature emerged as a significant barrier. This was mitigated by discussions on effective literature search strategies during the intervention, following which trainees reported improvement in their perceived ability to conduct literature searches (OR = 3.98, 95% CI = 1.143-13.89, p = 0.03) (Figure 3). The intervention also addressed trainee perspectives regarding limited opportunities and venues for presenting their scholarly activity. Trainees found the session helpful, significantly increasing their confidence in finding suitable venues for presenting their scholarly work (OR = 15.16, 95% CI = 4.21-54.54, p = 0.0001) (Figure 3).

## Discussion

Internal medicine residents face several barriers while engaging in scholarly activity, including inadequate training and workshops in literature search, deficiency in faculty mentorship, restricted financial support, lack of networking and collaboration, limited ideas about venues/conferences, and time constraints. Most survey respondents felt the need for more structured training or workshops significantly hindered their participation in scholarly activities. Many residents felt unprepared or unsure about initiating the process, including identifying suitable cases, research topics, and literature searches. These factors pose significant challenges among trainees in initiating, completing, and publishing scholarly work.

Our findings resonate with existing literature, often pointing to a formal research education deficiency within residency programs [2,3,5,6]. Our study demonstrated that a focused academic workshop on identifying impactful case reports, delivering oral presentations, and structuring scientific writing could overcome most of the barriers reported by trainees in the early stages of their research careers. Residents emphasized the importance of mentorship in guiding them through the research process, providing feedback on their work, and helping them navigate publication avenues. However, most teaching faculty members need more training in mentoring students and residents and often grapple with various clinical and nonclinical duties [3,7,8]. A study by Kichler et al. demonstrated that a mentor’s research productivity, particularly their publication output and acquisition of federally funded grants, significantly influenced residents’ success in finalizing a scholarly project [6]. The benefits of mentorship during residency training are well established [9,10]. Implementing effective mentorships could be addressed by promoting experienced faculty/chief resident-led journal clubs and research forums to enhance mentorship opportunities within the program.

The presentation of scholarly activity at regional and national meetings allows residents to engage in networking and collaboration. However, trainees often need more awareness of these venues and conferences. To address this gap, we introduced various platforms such as ACP, SGIM, SHM, and their timeline to residents where they could submit and present their scholarly work and engage in learning, communication, and networking. This initiative aimed to enhance residents’ confidence and presentation skills, foster critical thinking, and strengthen their CVs for potential fellowship applications. Our intervention effectively addressed these concerns, significantly improving trainees’ understanding of the measures to tackle networking and collaboration issues.

Financial constraints posed another challenge among residents, particularly regarding funding for research projects and conference presentations. Community-based programs like Sparrow Hospital may need help securing resources compared to larger academic institutions [6]. In the United States, residency programs are often allocated Continuing Medical Education funds to empower and encourage residents to pursue scholarly activities. Residents may benefit from program-led workshops on appropriate utilization of these funds. However, this cannot be generalized to training programs outside the United States, which may not have these provisions. “Green open access” journals, or journals requiring their readers to pay a fee to access the article, may be potential options for trainees to publish their work at a subsidized fee or even free of cost. Appropriate time management and balancing clinical and scholarly work was another challenge frequently encountered among trainees [3,6]. Strategies such as appropriately utilizing protected time for research and collaborating with other colleagues were highlighted during the intervention to address this issue [5].

Limitations

While our pilot study provides valuable insights into the effectiveness of targeted interventions in addressing barriers to scholarly engagement among internal medicine residents, several limitations should be acknowledged. This was a single-center study. Not all residents were present or obligated to participate in the intervention session, leading to a suboptimal response rate. We did not collect information on the respondents’ year of residency, which could have provided valuable insights into longitudinal trends and the impact of curricular changes.

Our survey was conducted among a small cohort of internal medicine residents, and the intervention was implemented for a short time, thus decreasing the overall power of the study. Other specialties outside of internal medicine were not explored in this small pilot study, which may have increased the generalizability of the study.

Our study focused on residents’ perceptions of case report writing and publication. Studies exploring the limitations and barriers of residents in engaging in original research work, systematic reviews, or meta-analyses may yield different and even more extensive findings.

## Conclusions

Our study highlights the various challenges internal medicine residents face when engaging in scholarly activity. Identifying these barriers and implementing specific interventions, such as interactive sessions, workshops, mentorships, and residency programs, would create a conducive environment that values and prioritizes meaningful research engagement. The study also emphasized the importance of developing a dedicated research curriculum. Institutional and program-specific support through financial means and protected scholarly time may improve research productivity and equip future physicians with an aptitude to practice research-based medicine.

## References

[1] (2023). Accreditation Council for Graduate Medical Education Residency Program Training Requirements. https://www.acgme.org/globalassets/pfassets/programrequirements/cprresi-dency_2023.pdf..

[2] Basu Ray I, Henry TL, Davis W, Alam J, Amedee RG, Pinsky WW (2012). Consolidated academic and research exposition: a pilot study of an innovative education method to increase residents' research involvement. Ochsner J.

[3] Tumilty H, Henning R, Obasi J, Pfeifer K, Bhandari S, Jha P (2020). Internal medicine residents’ perception of writing and presenting case reports. WMJ.

[4] Manring MM, Panzo JA, Mayerson JL (2014). A framework for improving resident research participation and scholarly output. J Surg Educ.

[5] Willett LL, Paranjape A, Estrada C (2009). Identifying key components for an effective case report poster: an observational study. J Gen Intern Med.

[6] Kichler K, Kozol R, Buicko J, Lesnikoski B, Tamariz L, Palacio A (2014). A structured step-by-step program to increase scholarly activity. J Surg Educ.

[7] Jha P, Thakur A, Klumb J, Bhandari S (2018). Perceptions of fourth-year medical students on writing and presenting case reports. Cureus.

[8] Rivera JA, Levine RB, Wright SM (2005). Completing a scholarly project during residency training. Perspectives of residents who have been successful. J Gen Intern Med.

[9] Joe MB, Cusano A, Leckie J (2023). Mentorship programs in residency: a scoping review. J Grad Med Educ.

[10] Crawford P, Seehusen D (2011). Scholarly activity in family medicine residency programs: a national survey. Fam Med.

